# Meglumine Antimoniate and Miltefosine Combined With Allopurinol Sustain Pro-inflammatory Immune Environments During Canine Leishmaniosis Treatment

**DOI:** 10.3389/fvets.2019.00362

**Published:** 2019-10-18

**Authors:** Marcos Ferreira Santos, Graça Alexandre-Pires, Maria A. Pereira, Cátia S. Marques, Joana Gomes, Jorge Correia, Ana Duarte, Lídia Gomes, Armanda V. Rodrigues, Alexandra Basso, Ana Reisinho, José Meireles, David Santos-Mateus, Maria Teresa Villa Brito, Luís Tavares, Gabriela M. Santos-Gomes, Isabel Pereira da Fonseca

**Affiliations:** ^1^Faculdade de Medicina Veterinária, CIISA–Centro de Investigação Interdisciplinar em Sanidade Animal, Universidade de Lisboa, Lisboa, Portugal; ^2^GHTM–Global Health and Tropical Medicine, Instituto de Higiene e Medicina Tropical (IHMT), Universidade Nova de Lisbon (UNL), Lisbon, Portugal

**Keywords:** canine leishmaniosis, peripheral blood mononuclear cells, lymph node, bone marrow, cytokine gene expression, meglumine antimoniate, miltefosine, allopurinol

## Abstract

Canine leishmaniosis (CanL) caused by *Leishmania infantum* is a zoonotic disease of global concern. Antileishmanial drug therapies commonly used to treat sick dogs improve their clinical condition, although when discontinued relapses can occur. Thus, the current study aims to evaluate the effect of CanL treatments in peripheral blood, lymph node, and bone marrow cytokine profile associated with clinical recovery. Two groups of six dogs diagnosed with CanL were treated with miltefosine combined with allopurinol and meglumine antimoniate combined with allopurinol (MT+A and MG+A), respectively. At diagnosis and after treatment, during a 3-month follow-up, clinical signs, hematological and biochemical parameters, urinalysis results and antileishmanial antibody titers were registered. Furthermore, peripheral blood, popliteal lymph node, and bone marrow samples were collected to assess the gene expression of IL-2, IL-4, IL-5, IL-10, IL-12, TNF-α, TGF-β, and IFN-γ by qPCR. In parallel, were also evaluated samples obtained from five healthy dogs. Both treatment protocols promoted the remission of clinical signs as well as normalization of hematological and biochemical parameters and urinalysis values. Antileishmanial antibodies returned to non-significant titers in all dogs. Sick dogs showed a generalized upregulation of IFN-γ and downregulation of IL-2, IL-4, and TGF-β, while gene expression of IL-12, TNF-α, IL-5, and IL-10 varied between groups and according to evaluated tissue. A trend to the normalization of cytokine gene expression was induced by both miltefosine and meglumine antimoniate combined therapies. However, IFN-γ gene expression was still up-regulated in the three evaluated tissues. Furthermore, the effect of treatment in the gene expression of cytokines that were not significantly changed by infection, indicates that miltefosine and meglumine antimoniate combined therapy directly affects cytokine generation. Both combined therapies are effective in CanL treatment, leading to sustained pro-inflammatory immune environments that can compromise parasite survival and favor dogs' clinical cure. In the current study, anti-inflammatory and regulatory cytokines do not seem to play a prominent role in CanL or during clinical recovery.

## Introduction

Leishmaniosis constitute a group of parasitic diseases of worldwide concern, that are considered by the World Health Organization as neglected tropical diseases (1). Canine leishmaniosis (CanL) caused by the intracellular protozoan *Leishmania infantum* is a zoonotic disease endemic to several southern European countries, including Portugal. In CanL, a wide range of non-specific clinical signs can be present (2), posing difficulties to a correct diagnosis. Previous studies differentiated sick dogs into symptomatic, oligosymptomatic and polysymptomatic (3–6) although more recently it has been proposed an improved system to stage dog's clinical condition (7, 8). This classification system takes into account the physical examination, clinicopathological abnormalities, anti-*Leishmania* antibody titer, and the evaluation of renal function according to the International Renal Interest Society guidelines (9). Other proposals also consider a first stage of exposed dogs as those living or that have lived in geographic regions in which the presence of vectors has been confirmed (10). CanL conventional treatments improve the dog's clinical condition, reducing skin parasite load and consequently the risk of *Leishmania* transmission. Although it is not definitively proved that treatment completely eliminates the parasite (11), and relapses are common when therapy is discontinued (3, 11, 12) it remains crucial to improve the efficiency of protocols used for CanL treatment. The main protocols for dog treatment usually include meglumine antimoniate (N-methylglucamine antimoniate), miltefosine (1-O-hexadecylphosphocholine), and allopurinol. Meglumine antimoniate is a pentavalent antimonial-based drug whose precise mechanism of action is not yet well-understood, but being considered a multifactorial drug with probable activity on parasite molecular processes, and influence in macrophage microbicide activity (13, 14). Miltefosine is an alkylphosphocholine compound able to induce apoptosis by mechanisms still not entirely clear (15–18). Allopurinol is a purine analog of adenosine nucleotide, which blocks RNA synthesis, inhibiting *Leishmania* growth (19). Up to date, meglumine antimoniate in combination with allopurinol is considered the first line of treatment in Europe (2), while miltefosine plus allopurinol has been the second line of treatment. However, miltefosine therapy has been gaining more attention (3–6), being recently authorized in 2017 for CanL treatment in Brazil (20), a highly endemic country for both canine and human leishmaniosis. Nevertheless, with the arising of more reports of drug resistance that lead to either therapeutic failure, unresponsiveness or relapse, whether it be in humans or dogs, a deeper understanding of the usual therapies is imperative (13, 15, 21, 22).

The immune response of dogs evidencing leishmaniosis clinical signs has been usually characterized by higher levels of specific antibodies, along with a type-2 T-helper (Th2) response associated with the expression of interleukin (IL)-4, IL-5, and IL-6 (23–25). On the contrary, protective immunity is thought to be dependent on a strong type-1 T-helper (Th1) response characterized by IL-2, IL-12, tumor necrosis factor (TNF)-α, and interferon (IFN)-γ production (23, 25). Furthermore, parasites may suppress host immunity by engaging regulatory T-cells (Treg) thus enabling the persistence of the infection (26), with one study showing clearance of *Leishmania* infection after depletion of Treg populations in mice (27). Moreover, higher expression of regulatory cytokines (IL-10, tumor growth factor [TGF-β]) associated with high parasite burden observed in dogs presenting clinical signs (28) suggest a non-negligible role of these cytokines in disease progression. To the best of our knowledge, there is no study defining the ideal approach to CanL treatment based on the knowledge of the immune response elicited by the different treatment protocols, and there is only one study analyzing more than one parasite target organ in non-treated CanL (29). Therefore, further studies are essential to clarify how treatments affect dogs' ability to develop a protective immune response or, on the contrary, to elicit immune suppression of effector cells. In the present study, the influence of two different treatment protocols on disease evolution of naturally infected dogs and on immune response was evaluated by assessing the clinicopathological changes, and the gene expression of pro-inflammatory (IL-2, IL-12, TNF-α, IFN-γ), anti-inflammatory (IL-4, IL-5) and regulatory (IL-10, TGF-β) cytokines in blood, popliteal lymph node and bone marrow during a 3-month period.

## Materials and Methods

### Dog Selection

**Figure 1:**
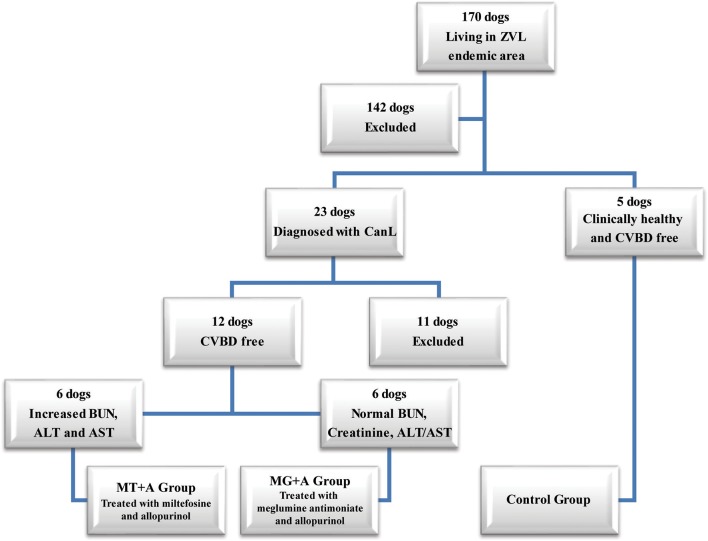
Flowchart representing the dog's selection process used in the current study. From a total of 170 dogs, living in an endemic area of zoonotic visceral leishmaniosis, two groups of dogs with canine leishmaniosis (CanL) were constituted, and were treated with either miltefosine in combination with allopurinol (MT+A) or with meglumine antimoniate in association with allopurinol (MG+A) along with one group of clinically healthy dogs (Control Group–CG). These dogs were negative for Canine Vector-Borne Diseases (CVBD). ALT, Alanine aminotransferase; AST, Aspartate aminotransferase; BUN, Blood urea nitrogen.

Twenty-three dogs with at least 1.5 years of age, weighing more than 5 kg, not having been vaccinated for CanL and diagnosed with CanL clinical stage I/II, according to the LeishVet Consensus Guidelines (7), and stage C in agreement to the Canine Leishmaniosis Working Group (CLWG) Guidelines (10) were selected from a total of 170 household dogs living in the zoonotic visceral leishmaniosis endemic area of the Metropolitan Area of Lisbon (Portugal). Twelve of those 23 dogs had not undergone any treatment in the last 8 months that could interfere with the immune response (such as antibiotic and corticosteroid therapy or administration of immunomodulators), and were negative for circulating pathogens potentially responsible of canine vector-borne diseases (CVBDs), were selected to participate in the current study. Five clinical healthy dogs not having been vaccinated for CanL, negative for *Leishmania* antibody test and CVBDs were also included in the present study as a control group (Figure 1). All dog owners gave written consent after being informed about the objectives of the study and every procedure, ensuring that clinical results were made available. Selected dogs include 13 males and 4 females of various breeds with ages ranging between 2 and 9 years and weight between 7.6 and 32.1 kg. Animal handling and sample collection procedures were done by the Veterinary team of the Teaching Hospital of the Faculty of Veterinary Medicine, University of Lisbon (Lisbon, Portugal). The present study followed the Council of the European Union Directive 86/609/EEC and was approved by the Ethics and Animal Welfare Committee of the Faculty of Veterinary Medicine, University of Lisbon.

### Experimental Design

**Figure 2:**
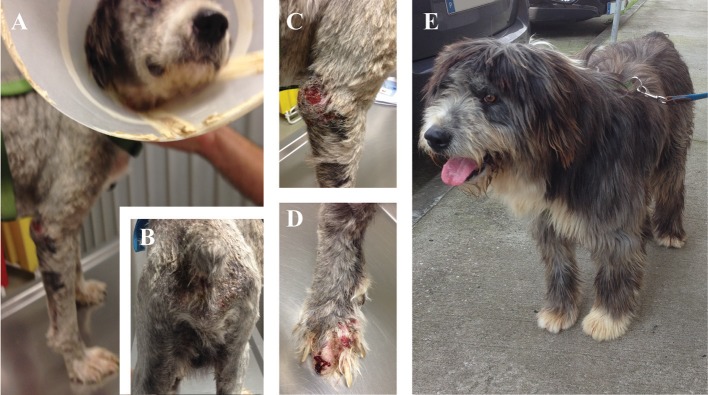
Clinical manifestations of a dog naturally infected with *Leishmania infantum*. **(A,B)** Dog presenting evident loss of weight, lethargy, cutaneous alopecia, and exfoliative dermatitis; **(C)** Ulcerative and hyperkeratosis lesions in the elbow of the front limb; **(D)** Onychogryphosis with severe bleeding; **(E)** Dog from the MT+A group 6 months after the diagnosis with full remission of clinical signs. Photos by Marcos Santos.

To reduce discomfort and ensure dog's well-being, the amount of sample collections and its periodicity were reduced to a minimum. Blood, popliteal lymph node, and bone marrow samples were collected from healthy (control group) and sick dogs prior the onset of treatment (Tp0) and then from sick dogs, 1 (Tp1), 2 (Tp2), and 3 months (Tp3) after the beginning of treatment. The samples collected from sick dogs at Tp0 were used, not only, to establish the baseline levels of cytokine mRNA accumulation, but also, for ethical reasons, to serve as controls of themselves, avoiding the need of an extra group of sick animals without any treatment. Treatment success was clinically and serological re-assessed 6 months after the initial diagnosis for each treated animal (Figure 2). Each dog was enrolled in one of the two treatment protocols (Figure 1), according to the following criteria:

- Dogs presenting increased blood urea nitrogen (BUN), creatinine and/or alanine aminotransferase (ALT), aspartate aminotransferase (AST), and UCP between 0.2 and 0.6, pointing to the possibility of developing hepatic and renal lesion were treated with miltefosine (Milteforan®, Virbac S.A, France; 2 mg/kg *per os, semel in die*–SID–for 4 weeks) in association with allopurinol (Zyloric®, Laboratórios Vitória, Portugal; 10 mg/kg, *per os, bis in die*–BID–for at least 6 months) (MT+A);
- Dogs presenting changes in biochemical and hematological parameters, serum proteins and UCP between 0.2 and 0.4 were treated with meglumine antimoniate (Glucantime®, Merial Portuguesa, Portugal; 100 mg/kg SID for 4 weeks) in association with allopurinol 10 mg/kg, *per os*, BID for at least 6 months (MG+A).

Deltamethrin-impregnated collars were applied to all dogs to prevent infections or re-infections during the current study and also in order to avoid *Leishmania* dissemination to sand flies. Blood samples were used for determination of hematological and biochemical parameters, and serological and molecular tests. Popliteal lymph node, bone marrow, and peripheral blood were used to examine cytokine gene expression. Urine samples were collected into sterilized containers for urinalysis and determination of protein/creatinine ratio (UPC).

### Sample Collection, Hematological and Biochemical Analysis, and Serological Tests

Peripheral blood (20 ml) was collected into syringes containing citrate phosphate dextrose adenine (CPDA-1, Medinfar Sorológico, Portugal). Popliteal lymph node aspirates were collected into syringes containing 0.8 ml of saline solution (0.9% NaCl) in order to avoid cell disruption and were then transferred to ethylenediaminetetraacetic acid (EDTA) tubes to avoid coagulation. After cutaneous anesthesia with a Xylocaine 10% Pump Spray (AstraZeneca, UK), bone marrow aspirates were collected from the distal area of the costal ribs, between the 9th and the 11th, into syringes containing 0.8 ml of saline solution. An additional 4 ml of blood was collected in EDTA tubes and dry tubes to be used for hematological (complete blood count), biochemical analysis (serum measurement of alanine aminotransferase (ALT), aspartate aminotransferase (AST), alkaline phosphatase, bilirubin, blood urea nitrogen (BUN), creatinine, inorganic phosphorus, calcium, sodium, potassium, chlorides), serum proteinogram electrophoresis, and CVBD screening. Peripheral blood samples were also used for the isolation of mononuclear cells. Popliteal lymph node and bone marrow were used for detection of *Leishmania* amastigote forms and isolation of mononuclear cells.

### *Leishmania* Screening

Serum samples were used for detection of anti-*Leishmania* antibodies by IFAT assay (*Leishmania-*Spot IF, BioMérieux, France) using *L. infantum* promastigotes as antigen and following the manufacturer's instructions. Samples were screened using an Olympus DP10 microscope (model BX50F, wavelength of 425 nm) and classified as positive if fluorescence was observed in promastigote cytoplasm or membrane at a serum dilution of 1:80 or higher. According to LeishVet (7) and the Canine Leishmaniosis Working Group (CLWG) guidelines (10), IFAT is a gold standard test for canine leishmaniosis and to evaluate possible relapses.

To test for the presence of *Leishmania* DNA, total genomic DNA was extracted from 200 μl of peripheral blood using the DNeasy® Blood and Tissue kit (QIAGEN®, Germany) according to the manufacturer's instructions. DNA amplification by qPCR was done in a total volume of 20 μl, comprising 10 μl of TaqMan® Gene Expression Master Mix (Applied Biosystems™, USA), 2 μl of ultra-pure water (Merck Millipore™ KGaA, Germany), 300 nM of forward and reverse primers for each set as well as 250 nM for each probe (Table 1) and 2 μl of target DNA. Reactions were carried out using the 7300 Real-Time PCR thermal cycler (Applied Biosystems™), with the following cycling conditions: 10 min at 95°C for AmpliTaq® Gold activation, followed by a total of 40 cycles of 15 s at 95°C and 1 min at 60°C. The positive control was constructed by cloning PCR fragments generated by the same primers into a pGEM®-T Easy Vector (Promega, USA), according to the manufacturer's instructions. Ligated fragments were transformed into JM109 competent cells and plasmid DNA was prepared using the QIAprep® Spin Miniprep Kit (QIAGEN®). The insert was sequenced using primers pUC/M13 (Promega) to ensure transformation stability. To exclude the presence of *Leishmania* amastigotes, lymph node and bone marrow slides were stained with Giemsa and observed by optical microscopy (Microscope Olympus CX31, using a 1,000× magnification).

**Table 1:** Primers and TaqMan probes used for hemoparasite screening.

**Target**	**Oligo**	**Oligonucleotide sequence (5^′^ → 3^′^)**	**Product size (bp)**	**References**
*Leishmania* (Kinetoplast)	Frw	GGAAGGTGTCGTAAATTCTGGAA	124	(30)
	Rev	CGGGATTTCTGCACCCATT		
	Probe^a^	AATTCCAAACTTTTCTGGTCCTCCGGGTAG		
*Ehrlichia* and *Anaplasma* (16S rRNA)	Frw	ACCTATAGAAGAAGTCCCGGCAA	100	(31)
	Rev	ACCTACGTGCCCTTTACGCCC		
	Probe^a^	GCAGCCGCGGTAATACGGAGGGGGC		
*Babesia* (18S rRNA)	Frw	ACCCATCAGCTTGACGGTAGGGT	97	(32)
	Rev	AGCCGTCTCTCAGGCTCCCT		
	Probe^b^	ACCGAGGCAGCAACGGGTAACGGGGA		
*Rickettsia* (OmpA)	Frw	AACCGCAGCGATAATGCTGAGTAGT	130	(33)
	Rev	CCCTGCAGAAGTTATCTCATTCCAA		
	Probe^b^	AGCGGGGCACTCGGTGTTGCTGCA		

*Presence of parasites in dog blood was evaluated by qPCR. Product base pair (bp) for each pathogen and primer references are also indicated. Frw, forward primer; Rev, reverse primer*.

a *Probe labeled with 6-FAM at the 5′-end and quenched with TAMRA at the 3′-end*.

b *Probe labeled with JOE at the 5′-end and quenched with TAMRA at the 3′-end*.

### CVBD Exclusion

Detection of antibodies against *Babesia, Anaplasma, Ehrlichia*, and *Rickettsia* was performed using commercial diagnostic tests (Megacor® MegaScreen, Austria, FLUOBABESIA *canis*–cut off 1:32; FLUOANAPLASMA *phagocytophilum*–cut off 1:50; FLUOEHRLICHIA *canis*–cut off 1:50; FLUORICKETTSIA *conorii*–cut off 1:40). The absence of *Babesia, Anaplasma, Ehrlichia*, and *Rickettsia* DNA was also evaluated by qPCR (Table 1) as previously described. To exclude the presence of *Dirofilaria immitis* microfilaria, blood samples were evaluated by Knott technique and parasite antigens were assessed by Witness® Dirofilaria kit (Zoetis, Portugal) according to the manufacturer's instructions.

### Cell Isolation

Dog peripheral blood was re-suspended in PBS (1:1 v/v), overlaid onto a 1:2 Histopaque®-1077 solution (Sigma-Aldrich, Germany) and centrifuged at 400 *g* for 30 min at 18°C. Mononuclear cells were harvested and washed in cold PBS (300 *g*, 10 min, 4°C), re-suspended in PBS, and the total volume adjusted to 2 × 10^7^ cells ml^−1^. Lymph node and bone marrow aspirates were centrifuged at 400 *g* (4°C) for 5 and 15 min, respectively, and re-suspended in 100 μl, with the total volume also adjusted for 2 × 10^7^ cells.ml^−1^. Then, 200 μl of peripheral blood mononuclear cells (PBMCs) and 100 μl of lymph node and bone marrow cell suspensions were centrifuged at 400 *g* (4°C) for 5 min, re-suspended in 600 μl of RLT Buffer (QIAGEN®) supplemented with β-mercaptoethanol and stored at −80°C until further use.

### mRNA Extraction and Reverse Transcription

Total RNA extracted from PBMCs, lymph node, and bone marrow cells, using RNeasy® Mini Kit (QIAGEN®) and QIAshredder® spin columns (QIAGEN®) was treated with DNase I Amplification Grade (Invitrogen™, USA) according to the manufacturer's instructions. For cDNA synthesis, 1 μg of purified RNA, presenting a 260/280 absorbance ratio ranging between 1.9 and 2.1 was denatured at 65°C for 5 min and reverse transcribed at 37°C for 60 min in a 30 μl final reaction mixture containing 6 μl of 5× M-MLV RT Buffer (Promega), 200 U/μl SCRIPT Reverse Transcriptase enzyme (Jena Bioscience, Germany), 500 μl dNTP Mix (Jena Bioscience), 1 μl of Oligo(dT)_18_ primers (Thermo Fisher Scientific Inc.™, EU), and 40 U/μl RiboLock RNase Inhibitor (Thermo Fisher Scientific Inc.™). cDNA samples were then heated at 95°C for 10 min for enzyme inactivation and stored at −20°C until further use.

### Cytokine Gene Expression

To evaluate the effect of treatment in pro-inflammatory, anti-inflammatory, and regulatory cytokines, the accumulation of mRNA encoding for IL-2, IL-4, IL-5, IL-10, IL-12, TNF-α, TGF-β, and IFN-γ was assessed by qPCR in PBMC, lymph node and bone marrow cell. cDNA amplification was conducted in a 20 μl final reaction mixture containing 10 μl of SYBR® Green PCR Master Mix (Applied Biosystems™), 80 nM of forward and reverse primers for each cytokine and for housekeeping gene β-actin (Table 2), 4 μl of ultra-pure water (Merck Millipore™ KGaA) and 2 μl of canine cDNA. Each sample amplification was performed in triplicate, using the following conditions: 10 min at 95°C for AmpliTaq® Gold activation followed by a total of 40 cycles (thermal profile for each cycle: 15 s at 95°C, 1 min at 60°C). An extra dissociation step was added to confirm the specificity of amplification by melting point analysis, and absence of non-specific products. External cDNA standards for all target cytokines and internal control used in every reaction were constructed as previously described. The concentration of standards was determined by measuring the OD at 260 nm followed by calculation of the corresponding copy number, and serial dilutions of resulting clones were used as standard curves, each containing a known amount of input copy number (39, 40). Copy numbers of target genes were normalized to the housekeeping gene β-actin, therefore correcting for minor variations in mRNA isolation and reverse transcription. Final results were expressed as the copy number of each cytokine per 1,000 copies of the housekeeping gene. Amplification efficiencies were >90%.

**Table 2:** Primers used for quantification of cytokine mRNA expression by qPCR.

**Target**	**Oligo**	**Oligonucleotide sequence (5^**′**^ → 3^**′**^)**	**Product size (bp)**	**References**
IL-2	Frw	GCATCGCACTGACGCTTGTA	86	(34)
	Rev	TTGCTCCATCTGTTGCTCTGTT		
IL-4	Frw	CATCCTCACAGCGAGAAACG	83	(35)
	Rev	CCTTATCGCTTGTGTTCTTTGGA		
IL-5	Frw	GCCTATGTTTCTGCCTTTGC	106	(36)
	Rev	GGTTCCCATCGCCTATCA		
IL-10	Frw	CAAGCCCTGTCGGAGATGAT	78	(37)
	Rev	CTTGATGTCTGGGTCGTGGTT		
IL-12p40	Frw	CAGCAGAGAGGGTCAGAGTGG	109	(34)
	Rev	ACGACCTCGATGGGTAGGC		
TNF-α	Frw	AATCATCTTCTCGAACCCCAAGT	74	(38)
	Rev	GGAGCTGCCCCTCAGCTT		
TGF-β	Frw	CAGAATGGCTGTCCTTTGATGTC	79	(35)
	Rev	AGGCGAAAGCCCTCGACTT		
IFN-γ	Frw	TCAACCCCTTCTCGCCACT	113	(36)
	Rev	GCTGCCTACTTGGTCCCTGA		
β-actin	Frw	ACGGAGCGTGGCTACAGC	62	(38)
	Rev	TCCTTGATGTCACGCACGA		

*bp, base pair; Frw, forward primer; Rev, reverse primer*.

### Data Analysis

An exploratory multivariate statistical analysis, specifically the Principal Component Analysis (PCA), was performed per tissue, on all datasets, in order to identify principal components accounting for the majority of the variation and graphically assess the separation between the healthy control, sick (Tp0) and treated dogs (Tp1, Tp2 and Tp3). This statistical analysis was performed using JMP version 14.3.0 (SAS Institute). Likewise, a K-Means Cluster analysis was also used to complement the previous PCA analysis and confirm grouping separation. In order to reduce the number of irrelevant or redundant variables and present a more robust model, a feature selection method was employed. Using the Predictor Screening tool from JMP the individual contribution of each variable was obtained, and the selected features were considered in the final models.

Statistical analysis between treatment groups was performed using GraphPad Prism software package version 8.0.1. Data normality was assessed using the Kolmogorov-Smirnoff test. Wilcoxon signed rank test was used to compare hematological and biochemical results in each dog treatment group between Tp0 and Tp3, with differences being considered significant when *p* < 0.05. The non-parametric Kruskal-Wallis Test (one-way ANOVA on ranks) with Dunn's *post hoc* test was used to evaluate differences in mRNA levels between treatment groups and the CG. The Repeated Measures ANOVA Test with Tukey's *post hoc* test was used to compare dogs at different time-points.

## Results

### Both Treatment Protocols Lead to Dog's Clinical Remission

Blood smears of dogs from MT+A, MG+A, and Control Group were all negative for CVBD causing agents. The dogs presented negative serology for *Babesia, Anaplasma, Ehrlichia*, and *Rickettsia*, and were negative for DNA detection of these parasites. Dogs were also negative in rapid immune migration for *D. immitis* antigen and microfilaria were absent in Knott technique.

**Figure 3:**
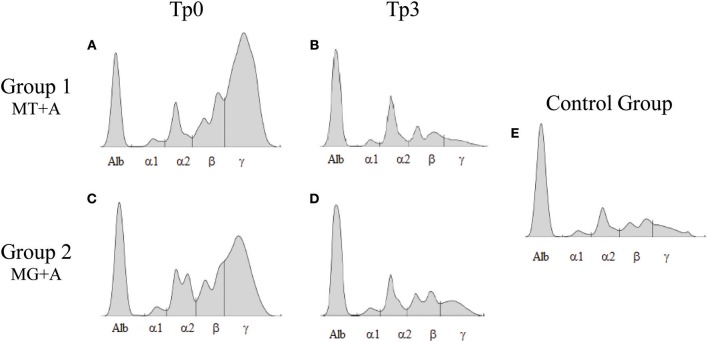
Serum protein electrophoresis of sick and treated dogs. Representative proteinograms of sick [Tp0; **(A)** MT+A; **(B)** MG+A], treated [Tp3; **(C)** MT+A; **(D)** MG+A] and clinically healthy dogs **(E)** are shown. Alb, Albumin; α1, α1-globulin; α2, α2-globulin; β, β-globulin; γ, γ-globulin.

Clinical signs observed in sick dogs at the beginning of the study (Tp0) included loss of body weight (Figures 2A,B), local/generalized lymphadenopathy, decreased/increased appetite, lethargy, mucous membrane pallor, polyuria/polydipsia, cutaneous alopecia, onychogryphosis (Figure 2D), hyperkeratosis, exfoliative-dermatitis, and erosive-ulcerative dermatitis (Figure 2C). Other clinical signs, such as epistaxis, lameness, and masticatory muscle myositis were also observed. No clinical signs were detected in dogs of the Control Group. Sick dogs showed also changes in hematological and biochemical parameters, including a mild decrease of hemoglobin values, mild erythropenia, lower hematocrit values, thrombocytopenia (Table 3), mild renal azotemia (Table 4), hyperglobulinemia with increased alpha 2 and gamma globulin fractions, and decreased values of alpha 1 and albumin/globulin ratio (Table 5, Figure 3). Dogs of group MT+A presented higher BUN values and an accentuated AST and ALT while dogs of the MG+A group exhibited BUN normal values and a slight increase in ALT and AST values (Table 4, Figure 3). Three dogs of group MT+A also showed creatinine values inferior to 1.4 mg/dL and mild proteinuria, presenting a urine protein:creatinine ratio (UPC) of 0.6. Control group dogs exhibited normal hematological and biochemical parameters, serum proteins, and urinalysis values. Lymph node and bone marrow smears of dogs from both MT+A and MG+A groups presented amastigote forms inside macrophages associated with lymphoid hyperplasia. Dogs from both groups showed anti-*Leishmania* antibody titers ranging between 1:80 and 1:320. No antileishmanial antibodies were detected in dogs from the Control Group (Table 6). One month after treatment (Tp1) dogs of MG+A exhibited higher vivacity and energy than dogs from MT+A. Three months after treatment onset (Tp3), both groups exhibited a successful recovery, showing remission of all clinical signs. Dogs from the MT+A group presented a significant recovery (*p* < 0.05) of BUN values to normal levels. AST and ALT quickly recovered to normal values in dogs of group MG+A (Table 4). Although presenting higher AST and ALT values, combined treatment of miltefosine and allopurinol promoted the decrease of AST and ALT in dogs from the MT+A group, albeit slower, with urinalysis values returning to normal. Dogs in MG+A group exhibited a normalization of the albumin globulin ratio 2 months after the beginning of treatment (Tp2) and 1 month later (Tp3) total protein and gamma globulin were within reference values. However, in dogs of the MT+A group the total protein and gamma globulin remained high and alpha 2 globulin normalized 3 months after the beginning of the treatment (Tp3) (Table 5, Figure 3). Three months after treatment onset (Tp3), MG+A dogs were negative for anti-*Leishmania* antibodies and, with the exception of one dog that had a titer of 1:320 group MT+A dogs were also negative. When re-evaluated 6 months after the initial diagnosis this positive dog was negative for antileishmanial antibodies (Table 6). Furthermore, amastigote forms were no longer observed in lymph node and bone marrow smears of dogs from both groups.

**Table 3:** Hemogram values exhibited by dogs of MT+A and MG+A groups.

**Hemogram**	**MT+A group (*****n*** **=** **6)**				**Tp0 vs. Tp3**	**MG+A group (*****n*** **=** **6)**				**Tp0 vs. Tp3**	**Control group (*n* = 5)**	**Reference interval**
	**Tp0**	**Tp1**	**Tp2**	**Tp3**		**Tp0**	**Tp1**	**Tp2**	**Tp3**			
RBC (× 10^6^/μl)	5.49 ± 1.32	5.30 ± 1.85	5.71 ± 1.07	5.87 ± 0.91	–	5.22 ± 0.55	5.54 ± 1.18	6.36 ± 0.65	6.82 ± 0.68	^*^	7.21 ± 1.05	5.5–8.5
Hemoglobin (g/dl)	12.32 ± 2.95	11.83 ± 3.55	13.08 ± 2.50	12.66 ± 3.24	–	11.24 ± 2.15	12.33 ± 2.59	14.25 ± 1.73	15.50 ± 1.26	^*^	16.26 ± 2.41	12–8
Hematocrit (%)	37.85 ± 10.89	35.45 ± 12.20	38.98 ± 7.60	37.44 ± 9.51	–	34.80 ± 6.09	37.38 ± 9.93	42.45 ± 5.86	45.27 ± 3.63	^*^	51.40 ± 7.29	37–55
MCV (μm^3^)	68.48 ± 4.13	67.15 ± 2.95	68.24 ± 2.80	68.72 ± 2.13	–	66.38 ± 7.04	67.12 ± 9.17	66.63 ± 4.71	66.53 ± 3.46	–	71.40 ± 1.69	60–74
MCH (pg)	22.60 ± 1.53	22.67 ± 1.35	22.92 ± 0.89	23.26 ± 0.54	–	21.02 ± 1.94	22.30 ± 1.48	22.42 ± 1.07	22.75 ± 1.25	–	22.54 ± 0.65	19.5–24.5
MCHC (g/dl)	33.10 ± 2.95	33.83 ± 2.65	33.70 ± 2.42	33.84 ± 0.78	–	32.32 ± 1.60	33.60 ± 3.51	33.72 ± 1.49	34.22 ± 1.28	–	31.62 ± 1.63	31–36
RDW (%)	13.16 ± 0.94	13.78 ± 0.95	13.88 ± 1.09	13.46 ± 1.44	–	13.43 ± 0.99	14.45 ± 2.35	13.08 ± 1.07	13.00 ± 0.97	–	12.34 ± 0.54	12–18
Leukocytes (× 10^3^/μl)	8.17 ± 2.98	7.83 ± 2.38	9.22 ± 2.13	7.76 ± 3.82	–	7.50 ± 2.47	8.62 ± 3.83	9.03 ± 2.90	9.55 ± 3.42	–	10.24 ± 3.18	6–17
Lymphocytes (× 10^3^/μl)	1.67 ± 0.51	2.23 ± 0.86	3.18 ± 1.64	2.96 ± 2.29	–	1.56 ± 0.85	2.12 ± 1.11	2.37 ± 0.81	2.22 ± 0.87	–	2.80 ± 0.70	1–4.8
Monocytes (× 10^3^/μl)	0.66 ± 0.25	0.48 ± 0.19	0.48 ± 0.22	0.40 ± 0.23	–	0.68 ± 0.24	0.67 ± 0.50	0.50 ± 0.26	0.47 ± 0.23	–	0.48 ± 0.20	0.2–2
Neutrophils (× 10^3^/μl)	5.60 ± 2.27	4.38 ± 1.35	4.92 ± 1.41	3.86 ± 1.45	–	5.11 ± 1.45	5.40 ± 2.71	5.77 ± 2.40	6.43 ± 2.95	–	5.96 ± 2.14	3–11.8
Eosinophils (× 10^3^/μl)	0.27 ± 0.28	0.70 ± 0.57	0.58 ± 0.40	0.52 ± 0.64	–	0.14 ± 0.21	0.42 ± 0.23	0.35 ± 0.19	0.38 ± 0.21	^*^	0.98 ± 0.40	0.1–1.3
Basophils (× 10^3^/μl)	0.02 ± 0.04	0.03 ± 0.05	0.04 ± 0.05	0.00 ± 0.00	–	0.02 ± 0.04	0.03 ± 0.05	0.07 ± 0.08	0.03 ± 0.05	–	0.06 ± 0.05	0–0.5
Platelets (× 10^3^/μl)	280.67 ± 133.4	233.83 ± 130.8	254.00 ± 150.8	235.00 ± 43.62	–	212.80 ± 133.5	246.50 ± 125.1	227.17 ± 60.38	222.50 ± 58.32	–	217 ± 25.84	200–500
MPV (μm^3^)	11.82 ± 2.94	12.82 ± 2.88	11.14 ± 2.27	11.82 ± 2.28	–	14.73 ± 3.14	11.82 ± 2.70	11.33 ± 2.56	10.73 ± 1.82	–	10.38 ± 1.28	5–15
Procalcitonin (%)	0.30 ± 0.14	0.28 ± 0.10	0.26 ± 0.13	0.26 ± 0.09	–	0.28 ± 0.15	0.23 ± 0.15	0.27 ± 0.08	0.25 ± 0.10	–	0.22 ± 0.04	0.2–0.5
PDW (%)	65.04 ± 11.24	64.33 ± 9.75	66.12 ± 11.74	64.62 ± 10.11	–	74.08 ± 3.95	57.98 ± 21.28	70.58 ± 8.48	73.87 ± 5.91	–	59.06 ± 5.03	40.6–65.2

*At diagnosis time (Tp0), and 1 (Tp1), 2 (Tp2) and 3 (Tp3) months after the beginning of the treatment. Blood samples of sick (n = 12) and healthy dogs (control group [CG], n = 5) were used to evaluate hemogram parameters. Reference values are also included. Wilcoxon signed rank text was used to compare between Tp0 and Tp3 in each treatment group*.

* *p < 0.05. MCH, Mean Corpuscular Hemoglobin; MCHC, Mean Corpuscular Hemoglobin Concentration; MCV, Mean Corpuscular Volume; MPV, Mean Platelet Volume; PDW, Platelet Distribution Width; RBC, Red Blood Cells; RDW, Red cell Distribution Width*.

**Table 4:** Biochemical parameters and urinalysis results exhibited by dogs of MT+A and MG+A groups.

**Biochemical parameters**	**MT+A group (*****n*** **=** **6)**				**Tp0 vs. Tp3**	**MG+A group (*****n*** **=** **6)**				**Tp0 vs. Tp3**	**Control group (*n* = 5)**	**Reference interval**
	**Tp0**	**Tp1**	**Tp2**	**Tp3**		**Tp0**	**Tp1**	**Tp2**	**Tp3**			
BUN (mg/dl)	60.35 ± 22.86	45.27 ± 35.79	33.23 ± 8.49	25.25 ± 7.04	^*^	26.03 ± 5.09	31.92 ± 3.84	34.50 ± 7.11	34.60 ± 6.82	^*^	36.33 ± 3.64	15-40
Creatinine (mg/dl)	1.20 ± 0.88	1.08 ± 0.68	1.19 ± 1.04	0.88 ± 0.62	–	0.55 ± 0.08	0.64 ± 0.12	0.87 ± 0.26	0.82 ± 0.27	–	0.92 ± 0.24	0.4-1.4
Total bilirubin (mg/dl)	0.05 ± 0.02	0.05 ± 0.01	0.06 ± 0.02	0.06 ± 0.04	–	0.04 ± 0.00	0.04 ± 0.00	0.05 ± 0.02	0.04 ± 0.00	–	0.06 ± 0.02	0.04-0.4
Direct bilirubin (mg/dl)	0.03 ± 0.01	0.03 ± 0.01	0.04 ± 0.02	0.02 ± 0.01	–	0.02 ± 0.01	0.02 ± 0.01	0.03 ± 0.01	0.02 ± 0.01	–	0.04 ± 0.01	0-0.3
Indirect bilirubin (mg/dl)	0.02 ± 0.01	0.02 ± 0.01	0.02 ± 0.01	0.04 ± 0.04	–	0.03 ± 0.01	0.02 ± 0.01	0.02 ± 0.01	0.02 ± 0.01	–	0.02 ± 0.02	0-0.3
AST (U/l)	62.80 ± 29.48	45.83 ± 12.12	46.17 ± 11.74	42.60 ± 17.62	–	49.25 ± 2.87	42.40 ± 13.79	58.50 ± 9.07	37.83 ± 17.36	–	40 ± 4.19	10-40
ALT (U/l)	93.83 ± 76.94	86.00 ± 42.70	78.33 ± 42.16	65.20 ± 40.53	–	44.17 ± 36.34	29.33 ± 16.19	30.33 ± 11.71	37.67 ± 16.11	–	40.25 ± 7.46	10-70
Alkaline phosphatase (U/l)	146.1 ± 166.4	131.78 ± 161.7	146.48 ± 243.8	30.05 ± 15.95	^*^	49.18 ± 15.34	38.95 ± 13.42	35.30 ± 7.10	35.28 ± 12.70	^*^	41.45 ± 29.56	20-200
Sodium (mmol/l)	145.60 ± 4.67	148.50 ± 8.76	146.50 ± 3.02	143.60 ± 3.21	–	142.25 ± 4.86	146.67 ± 2.34	146.67 ± 2.42	148.50 ± 3.56	–	146.75 ± 2.22	140-151
Potassium (mmol/l)	4.86 ± 0.79	4.70 ± 0.89	4.68 ± 0.49	4.54 ± 0.44	–	4.62 ± 0.34	4.59 ± 0.39	4.50 ± 0.27	4.40 ± 0.26	–	5.07 ± 0.53	3.4-5.4
Chloride (mmol/l)	112.80 ± 1.92	102.67 ± 13.94	113.50 ± 4.93	92.78 ± 51.56	–	108.00 ± 4.08	108.17 ± 9.35	104.17 ± 7.17	111.67 ± 6.02	–	113.00 ± 6.48	105-120
Calcium (mg/dl)	9.99 ± 0.51	9.54 ± 0.35	9.48 ± 0.72	9.08 ± 0.93	–	9.92 ± 0.42	9.93 ± 0.30	9.89 ± 0.32	10.14 ± 0.36	–	9.50 ± 1.57	9.5-12
Inorganic phosphorus (mg/dl)	4.96 ± 0.68	5.90 ± 2.87	4.35 ± 1.60	3.58 ± 0.92	–	3.95 ± 0.59	3.73 ± 0.67	3.35 ± 1.03	3.58 ± 1.25	–	4.60 ± 0.79	2.1-5
Biliary acids (μmol/l)	3.07 ± 2.11	3.22 ± 2.94	3.18 ± 3.34	3.36 ± 2.83	–	1.40 ± 0.25	3.54 ± 3.44	2.19 ± 1.28	1.72 ± 1.11	–	2.47 ± 1.34	1-10
**Urinalysis**												
Creatinine (mg/dl)	<1.4	<1.4	<1.4	<1.4	–	<1.4	<1.4	<1.4	<1.4	–	<1.4	<1.4
UPC	<0.2–0.6	<0.2–0.5	<0.2	<0.2	–	<0.2–0.4	<0.2–0.4	<0.2	<0.2	–	<0.2	<0.2

*At diagnosis time (Tp0), and 1 (Tp1), 2 (Tp2), and 3 (Tp3) months after the beginning of the treatment. Blood and urine samples of sick (n = 12) and healthy dogs [control group (CG), n = 5] were used to evaluate biochemical parameters and urinalysis. Reference values are also included. Wilcoxon signed rank text was used to compare between Tp0 and Tp3 in each dog group*.

* *p < 0.05. ALT, Alanine aminotransferase; AST, Aspartate aminotransferase; BUN, Blood Urea Nitrogen; UPC, Urine Protein Creatinine Ratio*.

**Table 5:** Serum proteins of dogs of MT+A and MG+A groups.

**Proteinogram**	**MT+A group (*****n*** **=** **6)**				**Tp0 vs. Tp3**	**MG+A group (*****n*** **=** **6)**				**Tp0 vs. Tp3**	**Control group (*n* = 5)**	**Reference interval**
	**Tp0**	**Tp1**	**Tp2**	**Tp3**		**Tp0**	**Tp1**	**Tp2**	**Tp3**			
Total protein (g/dl)	9.58 ± 1.55	7.70 ± 0.60	7.56 ± 1.18	7.60 ± 1.30	^*^	8.43 ± 1.46	7.66 ± 0.89	7.92 ± 1.30	6.85 ± 0.56	–	6.28 ± 0.59	5.5–7.5
Albumin (g/dl)	2.46 ± 0.84	2.48 ± 0.53	2.72 ± 0.24	2.56 ± 0.58	–	2.14 ± 0.50	2.50 ± 0.40	3.23 ± 1.01	3.05 ± 0.36	–	3.03 ± 0.40	2.26–4.3
Alpha 1 (g/dl)	0.22 ± 0.04	0.20 ± 0.00	0.20 ± 0.00	0.18 ± 0.04	–	0.20 ± 0.00	0.22 ± 0.04	0.27 ± 0.08	0.25 ± 0.05	–	0.28 ± 0.05	0.1–0.31
Alpha 2 (g/dl)	1.40 ± 0.25	1.32 ± 0.40	1.30 ± 0.35	1.22 ± 0.23	–	1.46 ± 0.23	1.40 ± 0.10	1.47 ± 0.35	1.20 ± 0.11	–	0.95 ± 0.06	0.5–1.1
Beta (g/dl)	1.78 ± 0.29	1.46 ± 0.09	1.64 ± 0.26	1.72 ± 0.42	–	1.58 ± 0.33	1.76 ± 0.13	1.73 ± 0.14	1.32 ± 0.31	–	1.38 ± 0.29	0.93–2
Gama (g/dl)	3.32 ± 2.36	2.26 ± 1.43	1.70 ± 1.44	1.92 ± 1.22	–	2.74 ± 1.52	1.82 ± 0.91	1.28 ± 0.26	1.10 ± 0.43	–	0.65 ± 0.19	0.3–1
Albumin: globulin ratio (%)	0.44 ± 0.29	0.50 ± 0.19	0.60 ± 0.17	0.56 ± 0.30	–	0.38 ± 0.13	0.50 ± 0.19	0.68 ± 0.17	0.80 ± 0.13	^*^	0.95 ± 0.13	0.6–1.1

*At diagnosis time (Tp0), and 1 (Tp1), 2 (Tp2) and 3 (Tp3) months after the beginning of the treatment. Blood samples sick (n = 12) and healthy dogs [control group (CG), n = 5] were used to evaluate serum proteins. Reference values are also included. Wilcoxon signed rank text was used to compare between Tp0 and Tp3 in each dog group*.

* *p < 0.05*.

**Table 6:** Anti-*Leishmania* antibody titers.

***Leishmania* antibody titer**	**Tp0**	**Tp1**	**Tp2**	**Tp3**	**Tp6**
Group 1 (*n* = 6)	1:80–1:320	<1:80–1:320	<1:80–1:160	<1:80	<1:80
Group 2 (*n* = 6)	1:80–1:320	<1:80–1:320	≤ 1:80	<1:80–1:320	<1:80
Control group (*n* = 5)	<1:80	<1:80	<1:80	<1:80	<1:80

*At diagnosis time (Tp0), and 1 (Tp1), 2 (Tp2), 3 (Tp3), and 6 (Tp6) months after the beginning of treatment, peripheral blood of sick (*n* = 12) and control dogs [control group (CG), *n* = 5] were collected and used to evaluated anti-*Leishmania* antibody titers by IFAT*.

*A cut-off of 1:80 was used*.

### Principal Component and Cluster Analysis Enable the Distinction Between Healthy and Sick Dogs

**Figure 4:**
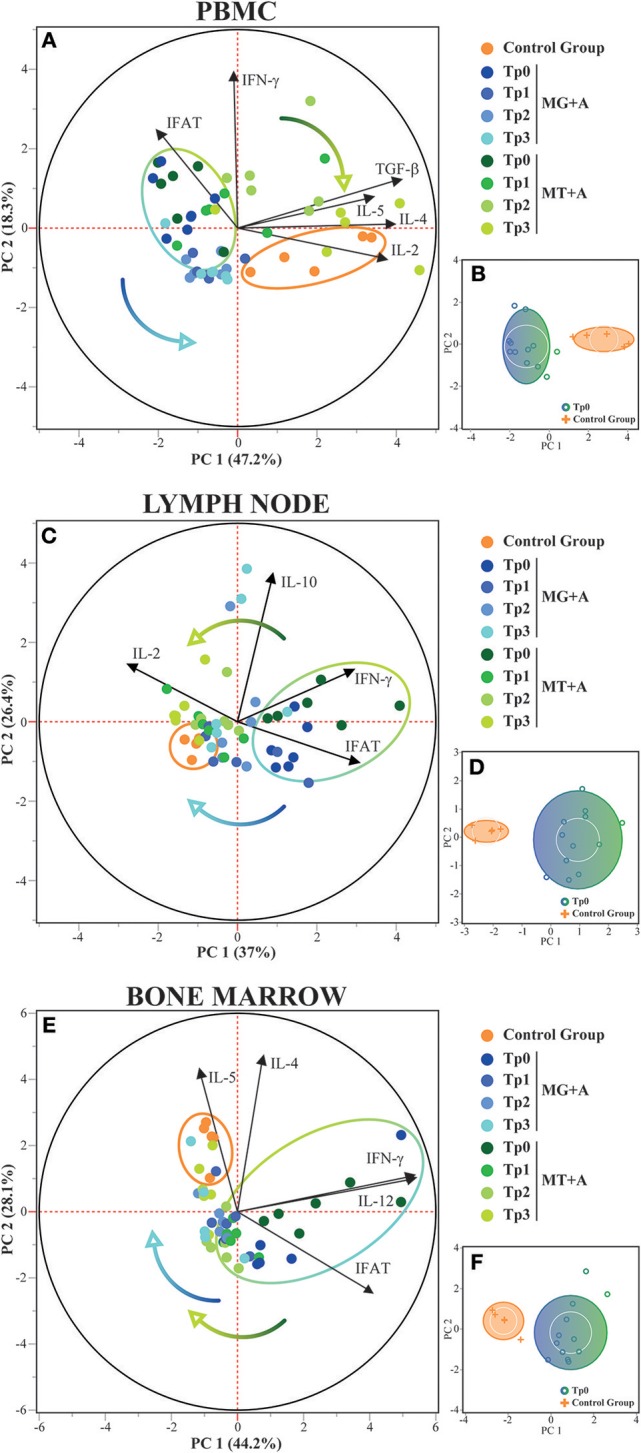
Principal Component and Cluster Analysis of cytokine expression in PBMC, lymph node and bone marrow. Principal component analysis was used to identify the first two principal components which explain 65.5, 63.4, and 72.3% for each respective tissue, of the variation in the dataset. **(A,C,E)** Biplot of score and loading plots showing the variables which load on the respective principal components. Control, MG+A, and MT+A groups are presented by different colored dots along all time-points, with the control group and the sick dogs (Tp0) delimited by their respective halo. Colored arrows show the transition of treated dogs over time. **(B,D,F)** Cluster analysis confirming the separation of healthy and sick dogs (Tp0) using the selected variables. PC, Principal Component.

Principal component analysis in PBMCs confirmed that healthy and sick dogs could be distinguished based on their expression of IFN-γ, IL-2, IL-4, IL-5, IL-12, and TGF-β along with IFAT results, with these features explaining 65.5% of the distribution (Figure 4A). In lymph node, PCA was also able to distinguish healthy and sick dogs based on the expression of IFN-γ, IL-2, and IL-10 along with IFAT results, with 63.4% of the distribution being explained by these variables (Figure 4C). For bone marrow the expression of IFN-γ, IL-4, IL-5, and IL-12 along with IFAT results, enabled the distinction between healthy and sick dogs, with these features explaining 72.3% of the distribution (Figure 4E). These results are also supported by cluster analysis (Figures 4B,D,F), with the formation of two separate groups. Dogs from both treatment groups could not be distinguished based on the selected features, but the transition from the sick dogs cluster toward the healthy dog cluster along the time-points can be observed in PBMC, lymph node, and bone marrow.

### *Leishmania* Infection Shapes Dogs' Cytokine Profile

**Figure 5:**
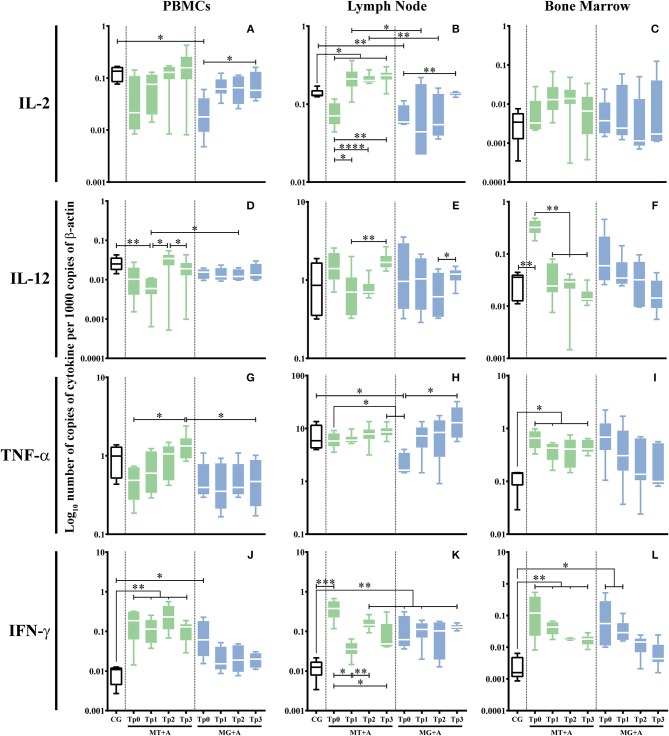
Pro-inflammatory cytokine gene expression in dogs treated with either MT+A or MG+A protocol along all time-points. IL-2 **(A–C)**, IL-12 **(D–F)**, TNF-α **(G–I)**, and IFN-γ **(J–L)** mRNA in PBMC **(A,D,G,J)**, lymph node **(B,E,H,K)** and bone marrow **(C,F,I,L)** cells of dogs from MT+A, MG+A, and Control Group (CG) was evaluated by qPCR. Results of 17 dogs and three replicates per sample are represented by box and whisker plot, median, minimum, and maximum values. The non-parametric Kruskal-Wallis Test (one-way ANOVA on ranks) with Dunn's *post hoc* test was used for statistical comparisons between treatments groups and the CG. The Repeated Measures ANOVA Test with Tukey's *post hoc* test was used for statistical comparisons inside each treatment group. *p < 0.05, **p < 0.01, ***p < 0.001, and ****p < 0.0001) indicate statistical significance.

Sick dogs (MT+A and MG+A) showed a significant accumulation of IFN-γ mRNA in cells of PBMC (*p*_MT+A_ = 0.0057; *p*_MG+A_ = 0.0425; Figure 5J), lymph node (*p*_MT+A_ = 0.001; *p*_MG+A_ = 0.0028; Figure 5K), and bone marrow (*p*_MT+A_ = 0.0097; *p*_MG+A_ = 0.0267; Figure 5L) when compared with clinically healthy dogs (CG). Bone marrow cells of dogs of MT+A showed a significant upregulation of IL-12 (*p* = 0.0059; Figure 5F) in comparison to control dogs. On the other hand, lymph node cells of sick dogs evidenced a significant reduction in IL-2 mRNA (*p*_MT+A_ = 0.0365; *p*_MG+A_ = 0.0068; Figure 5B). Dogs of MG+A group also showed a significant downregulation of IL-2 gene expression in PBMC (*p* = 0.0193; Figure 5A) and TNF-α in lymph node cells (*p* = 0.0186; Figure 5H). While dogs of MT+A group showed a significant upregulation of TNF-α gene expression in bone marrow cells (*p* = 0.0413; Figure 5I). No significant differences were found in gene expression of IL-12 by PBMC and lymph node cells, IL-2 by bone marrow cells and TNF-α by PBMC when compared to clinically healthy dogs.

**Figure 6:**
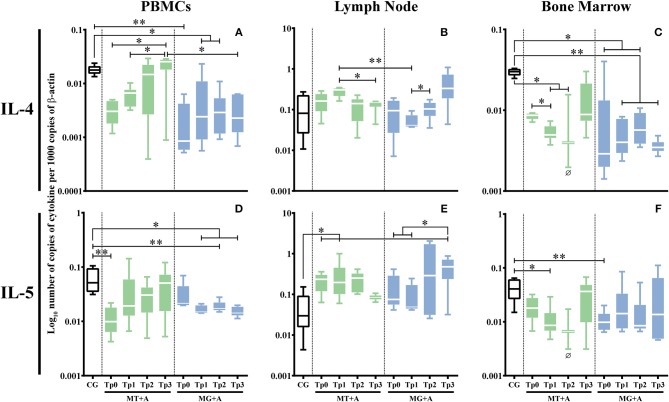
Anti-inflammatory cytokine gene expression in dogs treated with either MT+A or MG+A protocol along all time-points. IL-4 **(A–C)** and IL-5 **(D–F)** mRNA in PBMC **(A,D)**, lymph node **(B,E)**, and bone marrow **(C,F)** cells of dogs from MT+A, MG+A, and Control Group (CG) was evaluated by qPCR. Results of 17 dogs and three replicates per sample are represented by box and whisker plot, median, minimum, and maximum values. The non-parametric Kruskal-Wallis Test (one-way ANOVA on ranks) with Dunn's *post hoc* test was used for statistical comparisons between treatments groups and the CG. The Repeated Measures ANOVA Test with Tukey's *post hoc* test was used for statistical comparisons inside each treatment group. *p < 0.05, **p < 0.01 indicate statistical significance. Ø shows mRNA expression values of only three dogs.

PBMC (*p*_MT+A_ = 0.0662; *p*_MG+A_ = 0.0032; Figure 6A) and bone marrow (*p*_MG+A_ = 0.0138; Figure 6C) of sick dogs evidenced a significant down regulation of IL-4 gene expression in comparison to the CG. In lymph node cells, no significant differences were observed in the IL-4 gene expression. PBMC of sick dogs from MT+A group (*p* = 0.0031; Figure 6D) and bone marrow cells of dogs of group MG+A (*p* = 0.0082; Figure 6F) showed a statistically significant downregulation of IL-5 gene expression. Additionally, lymph node cells of MT+A showed a significant accumulation of IL-5 mRNA (*p* = 0.0235; Figure 6E).

**Figure 7:**
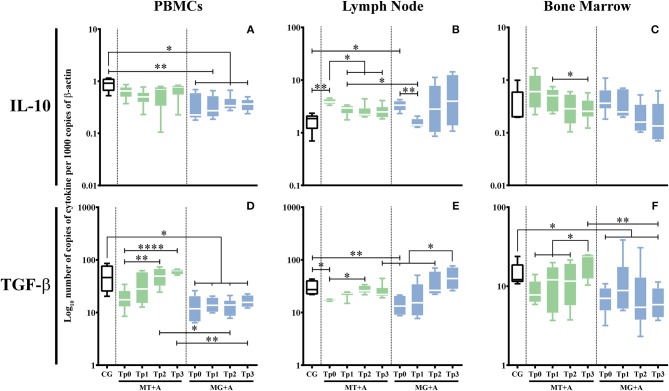
Regulatory cytokine gene expression in dogs treated with either MT+A or MG+A protocol along all time-points. IL-10 **(A–C)** and TGF-β **(D–F)** mRNA in PBMC **(A,D)**, lymph node **(B,E)**, and bone marrow **(C,F)** cells of dogs from MT+A, MG+A, and Control Group (CG) was evaluated by qPCR. Results of 17 dogs and three replicates per sample are represented by box and whisker plot, median, minimum, and maximum values. The non-parametric Kruskal-Wallis Test (one-way ANOVA on ranks) with Dunn's *post hoc* test was used for statistical comparisons between treatments groups and the CG. The Repeated Measures ANOVA Test with Tukey's *post hoc* test was used for statistical comparisons inside each treatment group. *p < 0.05, **p < 0.01, and ****p < 0.0001 indicate statistical significance.

A significant IL-10 downregulation in PBMC of MG+A (*p* = 0.0153; Figure 7A) and an upregulation in lymph node cells of sick dogs (*p*_MT+A_ = 0.0041, *p*_MG+A_ = 0.0112; Figure 7B). No significant differences in IL-10 gene expression were observed in bone marrow cells of sick dogs when compared with control dogs.

A significant reduction in the accumulation of TGF-β mRNA was observed in PBMC (*p*_MG+A_ = 0.0112; Figure 7D) and lymph node cells (*p*_MT+A_ = 0.0425; *p*_MG+A_ = 0.0057; Figure 7E) of sick dogs in relation to the CG. Bone marrow cells of MG+A group (Figure 7F) also showed a significant TGF-β downregulation (*p* = 0.0186).

Although there were differences between sick dogs, these results seem to indicate that *Leishmania* infection can shape the dogs' immune response by inducing IFN-γ upregulation while others pro-inflammatory (IL-2), anti-inflammatory (IL-4) and regulatory (TGF-β) cytokines were downregulated. Additionally, the modulation of TNF-α, a key player in macrophage activation, IL-5, which is involved in the differentiation of activated B lymphocytes into Ig-secreting plasma cells, and IL-10, a cytokine associated with immune regulation, seems to be tissue specific.

### Increased Gene Expression of Pro-inflammatory Cytokines Persists After Treatment With Miltefosine in Combination With Allopurinol

Gene expression of cytokines that were modified by infection was further evaluated along all time-points and studied tissues. In dogs treated with MT+A, bone marrow (Figure 5F) cells evidenced an IL-12 gene expression similar to CG 1 month after the beginning of treatment and throughout the study, pointing toward normalization. IFN-γ gene expression was still up-regulated in PBMC (*p*_Tp1_ = 0.0027, *p*_Tp2_ = 0.0023, *p*_Tp3_ = 0.0013; Figure 5J), lymph node (*p*_Tp2_ = 0.0061; Figure 5K) and bone marrow (*p*_Tp1_ = 0.0057, *p*_Tp2_ = 0.0061, *p*_Tp3_ = 0.0047; Figure 5L) cells during the observation period. In lymph node cells, there was a slight increase in IFN-γ mRNA accumulation at Tp2 when compared to Tp1 (*p*_Tp1_ = 0.0032). Nevertheless, a tendency to normalization was observed in bone marrow.

A significant high amount of IL-2 mRNA was found in lymph node cells at Tp3 (*p* = 0.0143) when compared to the CG (Figure 5B). In bone marrow cells, TNF-α gene expression persisted elevated (*p*_Tp1_ = 0.0303, *p*_Tp3_ = 0.0481) throughout all time-points (Figure 5I).

In PBMC, IL-4 gene expression recovered by Tp2 when compared with CG (Figure 6A), while in bone marrow a low accumulation of IL-4 mRNA (Figure 6C) was observed (*p*_Tp1_ = 0.0365, *p*_Tp2_ = 0.0420) throughout the study. Even so, at TP3 there was a slight upregulation of IL-4 gene expression, revealing a tendency to revert to normal values. Although a fluctuation of IL-5 gene expression was observed (Figure 6D), at Tp3 it reverts to values compared with CG. In lymph node cells, although IL-5 gene expression maintained increased at Tp1 (Figure 6E) when compared with CG (*p* = 0.0124), at Tp3 a trend to reduction in IL-5 mRNA accumulation points toward normalization.

IL-10 and TGF-β gene expression revealed a tendency to recuperation to normal values. Namely, IL-10 mRNA accumulation in lymph node (Figure 7B) was significantly decreased when compared to Tp0 (*p*_Tp1_ = 0.0338, *p*_Tp2_ = 0.0144, *p*_Tp3_ = 0.0409), along with a significant increase of TGF-β mRNA accumulation in PBMC (*p* < 0.0001) at Tp3 (Figure 7D) similar to CG and in lymph node at Tp2 (*p* = 0.0112) when compared with Tp0 (Figure 7E).

Despite the generalized tendency of treated dogs to achieve normal levels, the upregulation of pro-inflammatory cytokines (IFN-γ and IL-2) together with the trend to the normalization of anti-inflammatory (IL-4 and IL-5) and regulatory cytokines (IL-10 and TGF-β) point toward a persistent inflammatory immune response during the 3 months of treatment.

### Upregulation of IFN-γ Gene Expression Persists After Treatment With Meglumine Antimoniate Combined Therapy

Dogs treated with MG+A evidenced a normalization of IFN-γ gene expression in PBMC (Figure 5J). IFN-γ gene expression remained significantly higher (*p*_Tp1_ = 0.0029, *p*_Tp3_ = 0.0018) in lymph node cells in comparison to CG (Figure 5K). On the contrary, bone marrow cells (Figure 5L) showed a progressive decrease of IFN-γ mRNA. At Tp1 (*p* = 0.0425) the values were significantly increased when compared to the CG. However, by Tp2 and Tp3, IFN-γ gene expression lowered toward levels comparable to the CG. On the other hand, IL-2 (Figure 5B) and TNF-α (Figure 5H) gene expression in lymph node cells was similar to the CG. IL-2 at Tp1 in PBMC (Figure 5A) presented values similar to control dogs, with Tp3 having significant difference to Tp0 (*p* = 0.0425). The same was verified in lymph node, with IL-2 recovering to amounts comparable to CG by Tp3 (*p* = 0.0098). TNF-α (Figure 5H) in lymph node recovered to values similar to CG showing a significant difference when compared to Tp0 (*p* = 0.0451).

During treatment follow-up, IL-4 gene expression remained downregulated in PBMC (*p*_Tp1_ = 0.0219, *p*_Tp2_ = 0.0297) (Figure 6A) and in bone marrow cells (*p*_Tp1_ = 0.0068, *p*_Tp2_ = 0.0229, *p*_Tp3_ = 0.0013; Figure 6C) when compared with the CG. In PBMC, IL-5 gene expression was also downregulated (*p*_Tp1_ = 0.0199, *p*_Tp2_ = 0.0071, *p*_Tp3_ = 0.0343; Figure 6D). Despite a slight reduction in the accumulation of IL-5 mRNA in bone at Tp2, it was noticed a tendency to normalization (Figure 6F).

During treatment, accumulation of IL-10 (*p*_Tp1_ = 0.0076, *p*_Tp2_ = 0.0101, *p*_Tp3_ = 0.0108; Figure 7A) and TGF-β (*p*_Tp1_ = 0.0192, *p*_Tp2_ = 0.0235, *p*_Tp3_ = 0.0473; Figure 7D) mRNA was highly reduced in PBMC when compared to the CG. However, in lymph node cells, IL-10 (Figure 7B) and TGF-β (Figure 7E) gene expression was similar to control dogs. Nonetheless, at Tp3, a slight increase of TGF-β mRNA in lymph node was observed when compared to Tp2 (*p* = 0.0285). Despite bone marrow cells showed a normalization of TGF-β gene expression (Figure 7F), 2 months after treatment (Tp3) a significant decrease of TGF-β mRNA accumulation (*p* = 0.0453) was observed when compared to healthy dogs. *Leishmania* infected dogs treated with meglumine antimoniate in combination with allopurinol (MG+A) evidence a generalized tendency to achieve normal cytokine levels in the *Leishmania* host tissues evaluated in the current study. However, the persistent upregulation of IFN-γ gene expression associated with downregulation of IL-4, IL-5, IL-10, and TGF-β gene expression indicates the possible predominance of an inflammatory immune response. On the other hand, the slight increase of TGF-β at Tp3 in the lymph node can point toward the local activation of a regulatory immune response.

### The Activity of Miltefosine and Meglumine Antimoniate Combined Therapies Can Influence Cytokine Gene Expression

To estimate the influence of the drugs in cytokine generation, the cytokines that were not significantly altered by *Leishmania* infection were analyzed by comparing gene expression of sick (Tp0) and treated dogs (Tp1-Tp3).

After the first month of treatment (Tp1) with miltefosine in association with allopurinol (MT+A), PBMC evidenced a downregulation of IL-12 (*p* = 0.0025; Figure 5D) and IL-10 (Figure 7A), and a slight upregulation of IL-2 (Figure 5A) and TNF-α (Figure 5G). During the second and third month IL-2, TNF-α, and IL-10 showed a progressive upregulation, with IL-12 having an accentuated increase at Tp2 (*p* = 0.0411) and a slight decrease by Tp3 (*p* = 0.0233). Regarding lymph node cells, it was observed a considerable upregulation of IL-12 (Figure 5E) after Tp1 (*p* = 0.0062), along with a slight overexpression of TNF-α (Figure 5H) at Tp2 and Tp3 (*p* = 0.0138) time-points and a considerable gene expression of IL-4 (Figure 6B) at Tp1 followed by downregulation by Tp2 and Tp3 (*p* = 0.0410). In bone marrow, the treatment caused accumulation of IL-2 (Figure 5C) mRNA that persisted until Tp2, along with an increase of TGF-β (Figure 7F) that peaked at Tp3 (*p* = 0.0106). IL-5 (Figure 6F) mRNA levels showed a downregulation by Tp1 (*p* = 0.0343) that persisted until Tp2. A progressive downregulation of IL-10 (Figure 7C) was evident from Tp1 to Tp3 (*p* = 0.0452).

PBMC of dogs treated with MG+A showed a progressive downregulation of IL-12 (Figure 5D) from Tp0 to Tp2, followed by an increase at Tp3, and a progressive TNF-α (Figure 5G) increase from Tp0 that reached maximum values by Tp3. Lymph node cells presented IL-12 (Figure 5E) mRNA levels increased by Tp3 (*p* = 0.0484), exhibiting a slight and transitory downregulation of IL-4 (Figure 6B) levels at Tp1 (*p* = 0.0293) followed by a progressive upregulation that peaked at Tp3. IL-5 (Figure 6E) was slightly downregulated at Tp1 but showed an accentuate increase when meglumine antimoniate was discontinued (Tp2), with a slight decrease by Tp3 (*p* = 0.0344). Regarding bone marrow cells, a continuous decrease in IL-12 and TNF-α mRNA accumulation was noticed from Tp1 to Tp3. However, IL-2 gene expression presented an irregular pattern, suffering a downregulation at Tp2. When compared with Tp0, IL-10 presented a progressive downregulation until Tp3.

These findings indicate that MG+A directs the overexpression of cytokines in blood and lymph node and is possible that allopurinol plays a key role in enhancing cytokine generation. In the bone marrow, the drugs seem to downregulate cytokine gene expression. MT+A also seems to enhance cytokine gene expression. However, when miltefosine was discontinued, IL-4 in lymph node and IL-10 in the bone marrow became downregulated.

## Discussion

Progression of *L. infantum* infection is mainly dependent on the competence of the dog's immune system, which is related to inherent characteristics such as genetic background. Thus, the spectrum of clinical manifestations can range from subclinical infection to severe disease. During active disease, dog's immune response has been mainly characterized by a marked humoral immune response and specific immunosuppression of T lymphocyte proliferation (41). Despite being the major domestic reservoir of *L. infantum*, dogs have also intrinsic value, more notably a recognized social and affective role. Therefore, the use of therapies that can ensure a successful CanL treatment is highly required.

Several studies have pointed out the efficacy and faster recovery rate of dogs treated with meglumine antimoniate in combination with allopurinol (5, 19, 42, 43). Regarding the progress of hematological, biochemical, and urinary parameters, it is worth to emphasize that both combined therapies used in the current study were able to recover erythrocytes, hemoglobin, hematocrit, and UCP normal values, while leukocytes, neutrophils, creatinine, and albumin were within the reference intervals during the 3 months of treatment. Dogs evidencing less clinicopathological alterations, that were selected to be treated with meglumine antimoniate in combination with allopurinol, presented a fast recovery of hematological, biochemical and urinary parameters. Dogs showing more clinicopathological alterations, and which were treated with miltefosine in combination with allopurinol, took longer to reach normalization of those parameters.

Three months after CanL diagnosis (Tp3), both combined therapies were successful in promoting remission of clinical signs, recovering of hematological and biochemical normal values in all dogs and in restraining parasite infection since amastigotes were not found in the bone marrow and lymph node smears. Anti-parasite antibodies also diminished to non-significant titers in most of the dogs, with only one dog treated with MG+A taking more time to become negative (>3 months).

During CanL, *L. infantum* parasites are hosted in several organs of the reticuloendothelial system, having a widespread influence on the host's immune system. As previously reported (28, 39, 44), in CanL, IFN-γ gene expression is increased in parasite-host tissue prior to any treatment. Also in the current study PBMC, lymph node and bone marrow cells evidenced a pronounced generation of IFN-γ. Although such immune response is widely verified in many other studies, it also raised the question if this Th1 immune response is positively correlated with parasite control. Previous studies in experimentally infected hamsters and in humans suffering from visceral leishmaniosis have shown high parasite loads in Th1 environments, indicating an IFN-γ inability to confer protection (45, 46). Thus, the main consensus indicates that sick dogs express high levels of IFN-γ in *Leishmania*-target tissues, possibly directing a Th1 immune response against persistent infection.

The most studied tissue regarding cytokine expression during CanL is the peripheral blood, which in animals presenting clinical signs is characterized as having suppression of T cell mediated immunity and production of high levels of specific antibodies (24), as a consequence of a predominantly Th2 response with production of anti-inflammatory cytokines, such as IL-4 and IL-5 (23). In the present study, with the exception of high IFN-γ gene expression, peripheral blood IL-2, TGF-β, IL-4, and IL-5 of non-treated dogs were decreased, suggesting that *Leishmania* caused an overall lymphocyte deactivation, leading to unbalance of pro- and anti-inflammatory immune mediators. Still, taking into consideration that the peripheral blood is not the tissue of election for *L. infantum* replication and persistence (47, 48), along with possible natural genetic variability between dogs, it may be the reason why there is so much divergence between studies regarding cytokine expression in this tissue.

Despite most of CanL studies being focused in only one tissue, usually the peripheral blood, more and more studies consider that every single tissue affected by this parasite presents its own immune response (28, 39, 49, 50). IL-10 is a key regulatory cytokine that prevents excessive pathology. This cytokine can negatively regulate innate and adaptive immune responses by impairing the production of pro-inflammatory (e.g., IL-12, IL-2, IFN-γ, and TNF-α) and anti-inflammatory (IL-4 and IL-5) cytokines, restraining T cell activity in lymph nodes and limiting tissue inflammation. In CanL, the lymph node is reported as having a predominantly Th1 immune response (39). Besides this, a true consensus has not been established, with studies showing higher expression of Th1 cytokines, like IFN-γ and TNF-α (51), in pre-scapular lymph nodes of dogs without external clinical signs and lower parasite burden, pointing toward a possible role of these cytokines in controlling parasite replication. In contrast, dogs presenting clinical signs showed no expression of IL-4 and IL-12, but high levels of immunosuppressor cytokines like IL-10 and TGF-β (51), posing a role in disease progression. In the current study, the lymph node of dogs with CanL seems to evidence a mixed Th1/Treg immune response with low IL-2, but high IL-12 and IFN-γ, along with down expression of TGF-β but over expression of IL-10, pointing toward a balance between the differentiation of IFN-γ mediated inflammatory response and a regulatory immune response that could favor parasite persistence.

Considering the cytokine expression in bone marrow of dogs with CanL, to our best knowledge, there are only a few documented studies (28, 39, 44), which report this tissue as a predominantly Th1 environment that tends to develop high parasite loads, characterized by an increased expression of IFN-γ and TNF-α and low to no detection of IL-10, along with lower expression of IL-4 (28, 44). In the current study, bone marrow cells of sick dogs also evidence IFN-γ overexpression and low expression of IL-4, IL-5, and TGF-β pointing to a predominantly pro-inflammatory immune response. Furthermore, the PCA and cluster analysis reinforce that each tissue presents a distinct cytokine pattern of response to infection, confirming previous reports (39). Furthermore, infection level also seems to influence local cytokine gene expression, namely TNF-α, that points toward a diminished generation of this cytokine in lymph node cells of dogs presenting less clinicopathological signs (MG+A), and overexpression in bone marrow cells of dogs with severe clinicopathological signs (MT+A). TNF-α together with IFN-γ induce the upregulation of inducible nitric oxide synthase (iNOS) by macrophages, directing L-arginine oxidation and nitric oxide (NO) production (52). NO is a powerful oxidative molecule that mediates parasite killing. Thus, the hypothesis that TNF-α can be a biomarker of CanL severity needs to be further investigated. Furthermore, IL-5, a cytokine linked to growth and differentiation of B cells, evidenced to be over-expressed in lymph node cells of dogs presenting more clinicopathological signs (MT+A). These findings point to a higher B cell activation in lymph node. The over expression of IL-12 in bone marrow cells of dogs exhibiting more clinicopathological signs (MT+A), a signaling pathway cytokine that prime naïve T cells to differentiate into Th1 cells, supports the possible establishment of a Th1 cell population.

By analyzing the peripheral blood, popliteal lymph node and bone marrow along the course of two of the most used CanL protocol treatments, the current study shows evidence of a higher IFN-γ generation during the 3 months of follow up of dogs treated with MT+A. Furthermore, lymph node cells also exhibited a TNF-α overexpression, suggesting that there are conditions for macrophage activation and parasite inactivation, and increased generation of IL-2, indicating a possible lymphocyte proliferation. These findings indicate that miltefosine associated therapy does not promote reduction of pro-inflammatory immune response, but, induces the normalization of anti-inflammatory IL-4 and IL-5 and of immune-suppresor TGF-β in mononuclear blood cells, of immune-suppressor IL-10 in lymph node and of IL-5, TGF-β and pro-inflammatory IL-12 in bone marrow.

MG+A lead to the normalization of the proinflammatory immune response, restoring IFN-γ and IL-2 expression levels in blood cells, IL-2, IL-12, and TNF-α in lymph node and IFN-γ in the bone marrow. Although showing some instability, IL-5 tends to normal values in the bone marrow. Treatment also seems to induce the normalization of immunosuppressor cytokines in the lymph node. However, the continuous overexpression of IFN-γ in lymph node cells points toward the maintenance of a local inflammatory response despite the drug activity in promoting the remission of clinical signs, and the rise of IFN-γ gene expression in mononuclear blood cells 1 month post-treatment suggests the predomination of a Th1 immune response. On the other hand, IL-4 and IL-5 stay downregulated in mononuclear blood cells as well as IL-10 and TGF-β indicating the inhibition of Th2 and Treg immune response even during dogs' clinical improvement. In bone marrow as well, treatment did not induce the normalization of IL-4 gene expression.

The effect of drug therapies used in the current study in cytokine gene expression was investigated in the cytokines that were not significantly affected by parasite infection (Tp0). Although combined therapies seem to have similar outcomes, it was not possible to find a distinctive pattern, exhibiting cytokine, and tissue dependent effects. The drug activity possibly empowered by free parasite antigens seems to favor mainly cytokine generation.

The current study enables a close overview of the effect of the two most used anti-leishmanial therapies, miltefosine, and meglumine antimoniate in association with allopurinol, in reversing CanL progression on naturally infected dogs, including clinical signs remission, normalization of hematological, biochemical and urinary parameters, and IFAT seroconversion. Both combined therapies are effective in CanL treatment, favoring clinical recovery of all dogs and the overexpression of pro-inflammatory cytokines, pointing toward the persistence of inflammatory immune environments that can direct parasite inactivation at least during the initial 3 months of treatment. The current study also demonstrates that anti-inflammatory and regulatory cytokines do not seem to play a key role in CanL immune response. Furthermore, the combined therapies also appear to play a direct role in cytokine generation. These are relevant findings, since both are two of the most used protocols in the treatment of this zoonotic parasitosis, the evolution of the cell-mediated immune response generated while under these specific treatments should be further studied. With the recent implementation of miltefosine for CanL treatment in Brazil, an extremely endemic country for canine and human leishmaniosis, it becomes a subject of ensuring the best for the reinforcement of Public Health protection.

## Data Availability Statement

The raw data supporting the conclusions of this manuscript will be made available by the authors, without undue reservation, to any qualified researcher.

## Ethics Statement

The animal study was reviewed and approved by Ethics Committee and Animal Welfare (CEBEA–Comissão de Ética e Bem-Estar Animal) of the Faculty of Veterinary Medicine, University of Lisbon. Written informed consent was obtained from the owners for the participation of their animals in this study.

## Author Contributions

GS-G, CM, MS, and IF conceived and designed the study. MS, CM, MP, JG, JC, AB, AR, JM, and IF collected samples. MS, LG, and IF processed samples and did subsequent microscopic, molecular, and serological tests. MS and CM conducted the experiments. MS, GS-G, IF, and MB analyzed the data. MS and GS-G conducted statistical analysis. MS, GS-G, and IF drafted the manuscript. GS-G, IF, GA-P, MB, AD, LT, AVR, MB, and DS-M made in depth reviews of the manuscript. All authors read and approved the final manuscript.

### Conflict of Interest

The authors declare that the research was conducted in the absence of any commercial or financial relationships that could be construed as a potential conflict of interest.

## Abbreviations

ALT: Alanine aminotransferase
AST: Aspartate aminotransferase
BID: *bis in die*
BUN: Blood urea nitrogen
CanL: Canine leishmaniosis
cDNA: Complementary DNA
CG: Control Group
CLWG: Canine Leishmaniasis Working Group
CPDA-1: Citrate phosphate dextrose adenine
CVBD: Canine vector-borne disease
DNA: Deoxyribonucleic acid
EDTA: Ethylenediaminetetraacetic acid
IL: Interleukin
IFAT: Indirect Fluorescent Antibody Test
IFN-γ: Interferon gamma
MCH: Mean corpuscular hemoglobin
MCHC: Mean corpuscular hemoglobin concentration
MCV: Mean corpuscular volume
MG+A: Meglumine antimoniate with Allopurinol
MPV: Mean platelet volume
MT+A: Miltefosine with Allopurinol
PBMC: Peripheral Blood Mononuclear Cell
PCA: Principal Component Analysis
PDW: Platelet distribution width
qPCR: Real-time PCR
RDW: Red cell distribution width
RNA: Ribonucleic acid
SID: *semel in die*
Th1: Type-1 T-Helper
Th2: Type-2 T-Helper
TGF-β: Transforming growth factor beta
TNF-α: Tumor necrosis factor alpha
Treg: Regulatory T-cells
UPC: Urine Protein Creatinine ratio.
